# Dysregulation of Placental Functions and Immune Pathways in Complete Hydatidiform Moles

**DOI:** 10.3390/ijms20204999

**Published:** 2019-10-10

**Authors:** Jennifer R. King, Melissa L. Wilson, Szabolcs Hetey, Peter Kiraly, Koji Matsuo, Antonio V. Castaneda, Eszter Toth, Tibor Krenacs, Petronella Hupuczi, Paulette Mhawech-Fauceglia, Andrea Balogh, Andras Szilagyi, Janos Matko, Zoltan Papp, Lynda D. Roman, Victoria K. Cortessis, Nandor Gabor Than

**Affiliations:** 1 Department of Obstetrics and Gynecology, Keck School of Medicine, University of Southern California, Los Angeles, CA 90033, USA; jennyrenaeking@gmail.com (J.R.K.); koji.matsuo@med.usc.edu (K.M.); lroman@usc.edu (L.D.R.); 2 Department of Preventive Medicine, University of Southern California, Los Angeles, CA 90033, USA; melisslw@usc.edu; 3 Systems Biology of Reproduction Research Group, Institute of Enzymology, Research Centre for Natural Sciences, H-1117 Budapest, Hungary; heteysz@gmail.com (S.H.); peter0kiraly@gmail.com (P.K.); toth.eszter@ttk.hu (E.T.); balogh.andrea@ttk.hu (A.B.); szilagyi.andras@ttk.hu (A.S.); 4 Department of Pathology, Keck School of Medicine, University of Southern California, Los Angeles, CA 90033, USA; antonio.castaneda@osumc.edu (A.V.C.); pfauceglia@hotmail.com (P.M.-F.); 5 First Department of Pathology and Experimental Cancer Research, Semmelweis University, H-1085 Budapest, Hungary; krenacst@gmail.com; 6 Maternity Private Clinic of Obstetrics and Gynecology, H-1126 Budapest, Hungary; hupuczi.petronella@maternity.hu (P.H.); pzorvosihetilap@maternity.hu (Z.P.); 7 Department of Immunology, Institute of Biology, Eotvos Lorand University, H-1117 Budapest, Hungary; matko@elte.hu; 8 Department of Obstetrics and Gynecology, Semmelweis University, H-1088 Budapest, Hungary

**Keywords:** choriocarcinoma, hydatidiform mole, galectin, gestational trophoblastic disease, placental-specific gene, systems biology, trophoblast differentiation

## Abstract

Gene expression studies of molar pregnancy have been limited to a small number of candidate loci. We analyzed high-dimensional RNA and protein data to characterize molecular features of complete hydatidiform moles (CHMs) and corresponding pathologic pathways. CHMs and first trimester placentas were collected, histopathologically examined, then flash-frozen or paraffin-embedded. Frozen CHMs and control placentas were subjected to RNA-Seq, with resulting data and published placental RNA-Seq data subjected to bioinformatics analyses. Paraffin-embedded tissues from CHMs and control placentas were used for tissue microarray (TMA) construction, immunohistochemistry, and immunoscoring for galectin-14. Of the 14,022 protein-coding genes expressed in all samples, 3,729 were differentially expressed (DE) in CHMs, of which 72% were up-regulated. DE genes were enriched in placenta-specific genes (OR = 1.88, *p* = 0.0001), of which 79% were down-regulated, imprinted genes (OR = 2.38, *p* = 1.54 × 10^−6^), and immune genes (OR = 1.82, *p* = 7.34 × 10^−18^), of which 73% were up-regulated. DNA methylation-related enzymes and histone demethylases were dysregulated. “Cytokine–cytokine receptor interaction” was the most impacted of 38 dysregulated pathways, among which 17 were immune-related pathways. TMA-based immunoscoring validated the lower expression of galectin-14 in CHM. In conclusion, placental functions were down-regulated, imprinted gene expression was altered, and immune pathways were activated, indicating complex dysregulation of placental developmental and immune processes in CHMs.

## 1. Introduction

Gestational trophoblastic disease (GTD) is characterized by abnormal trophoblastic proliferation and includes hydatidiform mole (complete and partial) and gestational trophoblastic neoplasia (invasive mole, choriocarcinoma, placental site trophoblastic tumor, and epithelioid trophoblastic tumor) [1,2]. Diagnosis relies on clinical presentation and histologic assessment, and treatment is aimed at uterine evacuation with chemotherapy typically reserved for gestational trophoblastic neoplasia (GTN) [2,3]. Post-molar human chorionic gonadotropin (hCG) monitoring is an essential part of management for evaluating the development of GTN, which follows complete hydatidiform moles (CHMs) in ~15–20% of cases [4]. Although GTN is considered among the most curable of all solid tumors with cure rates over 90%, unrecognized and misdiagnosed GTD can result in unnecessarily increased maternal morbidity and mortality [3].

The reported incidence of GTD varies by geographic location, race or ethnicity, maternal age, and histopathologic subtype. Hydatidiform mole reportedly complicates up to two per 1000 pregnancies in Southeast Asia and Japan, nearly twice that proportion reported in North America, Australia, New Zealand, and Europe [5]. This geographic variation has been partially attributed to racial and ethnic differences, as the prevalence of GTD is elevated in American Indians, Asians, and Hispanics across the world [6,7]. Asian and American Indian women have also been shown to have more aggressive disease, with increased risk of developing GTN compared to other ethnic groups [8,9]. Extremes of maternal age are also correlated with higher rates of CHMs, with an increased incidence among women over 40 years of age and under 20 years of age [10]. Beyond maternal age and ethnicity, prior GTD is the strongest risk factor for GTD, with an incidence of 1.3% [11].

These described risk factors (race/ethnicity and prior GTD) are in accord with an underlying genetic etiology of GTD [1,2,12]. The pathophysiology of hydatidiform moles involves overrepresentation of the paternal genome. The biparental diandric triploid karyotype of partial moles accords with dispermic fertilization, while androgenetic diploid karyotype of most CHMs is consistent with monospermic or dispermic fertilization. Recently, some women with recurrent androgenetic CHMs were shown to have bi-allelic deleterious mutations in *MEI1* (meiotic double-stranded break formation protein 1), *TOP6BL* (type 2 DNA topoisomerase 6 subunit B-like), and *REC114* (meiotic recombination protein REC114), leading to meiotic double-strand break formation and extrusion of all maternal chromosomes [13]. Absence of maternal imprinting of gene expression in hydatidiform moles has also been observed in the rare biparental hydatidiform moles due to *NLRP7* (NLR family pyrin domain containing 7) or *KHDC3L* (KH domain containing 3 like) mutations, suggesting a common endpoint of pathogenesis [12,14,15]. However, for the more common sporadic CHMs, little is known regarding mechanisms responsible for either pathogenesis or progression to GTN.

The few targeted gene expression studies on molar tissue and a recent meta-analysis of these studies showed that the main genes differentially expressed (DE) in molar tissues may be those involved in villous trophoblast differentiation [16]. However, these findings were based on a limited set of molecules, and these studies mostly targeted placenta- or trophoblast-specific transcripts that were known to be differentially expressed during trophoblast differentiation. A more comprehensive approach to identifying genes and pathways involved in the development of molar disease would be a genome-wide gene expression analysis using either microarrays or RNA-Seq, followed by protein-level validation of DE transcripts.

We sought to implement such a high-dimensional and systems biology approach, similar to that used in our recent study on the pathophysiological processes in preeclampsia [17], to gain more in-depth insight into CHM pathogenesis at RNA and protein levels. This high-dimensional, agnostic study is the first to evaluate gene expression levels in CHMs using RNA-Seq followed by protein level validation of selected DE transcripts by immunostaining of tissue microarrays (TMA) and immunoscoring. The aim of our study is to identify genes with expression levels that differ in molar tissue from CHMs in comparison to placental chorionic tissue from uncomplicated pregnancies at similar stages of gestation. More complete understanding of the molecular pathways perturbed in CHMs may inform future efforts to improve procedures for early diagnosis and prognostication.

## 2. Results

### 2.1. The Transcriptome of First Trimester Placentas and CHMs

To evaluate absolute gene expression levels, mean expression values were calculated for both groups from RNA-Seq count data by normalizing for housekeeping genes. The highest expression in first trimester placentas was mostly detected for genes with placenta-specific or predominant placental expression [17,18,19]. Indeed, the 20 most highly-expressed genes (Table 1) included genes previously shown to have predominant placental (*n* = 2) or placenta-specific (*n* = 12) expression and unique placental functions in humans. These encode hormones (*CGA*, chorionic gonadotrophin subunit alpha, *CSH1* and *CSH2*, chorionic somatomammotropin hormone 1 and 2), an estrogen synthesizing enzyme (*CYP19A1*, cytochrome P450 family 19 subfamily A member 1), proteases (*ADAM12*, ADAM metallopeptidase domain 12; *KISS1*, kisspeptin-1; *PAPPA* and *PAPPA2*, pregnancy-associated plasma protein A and A2; *TFPI2*, tissue factor pathway inhibitor 2), and immune proteins (*PSG3* and *PSG4*, placenta-specific glycoprotein 3 and 4).

In CHMs, the overall median gene expression levels were ~13% higher than in normal placentas (control placentas: 337.7 vs. CHMs: 382.8). However, placenta-specific transcript levels were 23% lower in CHMs than in normal placentas (placentas: 3,729.6 vs. CHMs: 3,044.3), reflected in a lower number of placenta-specific genes (*n* = 8) among the 20 most highly-expressed transcripts (Table 2). In turn, the 20 most abundant transcripts in CHMs encode proteins with immune, hormone, and oxygen transport functions (*FSTL3*, follistatin-like 3; *HBA2*, hemoglobin-alpha 2; *HBB*, hemoglobin-beta; *IGF2*, insulin-like growth factor 2; *LEP*, leptin; *PAEP*, progestogen-associated endometrial protein).

### 2.2. Differential Gene Expression in CHMs

**Figure 1:**
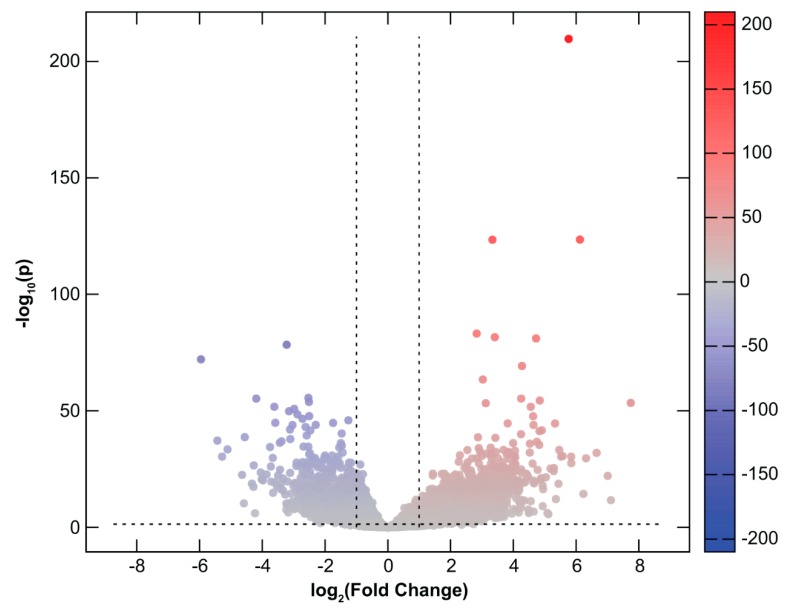
Differential gene expression in complete hydatidiform moles. All 14,022 expressed protein-coding genes are represented in terms of their measured differences in transcript abundance (x-axis) and the significance of the difference (y-axis) on a volcano plot. The significance is represented as negative log (base 10) of the adjusted *p*-value so that more significant differences in expression are plotted higher on the y-axis. Dotted lines represent the thresholds used to select the differentially expressed (DE) genes: <−1 and >1 for the magnitude of differential expression and pFDR <0.05 for statistical significance. According to these criteria, of the 3,729 DE transcripts, 2,667 were up-regulated (depicted with red), while 1062 were down-regulated (depicted with blue) in molar tissues.

Among transcripts of 14,022 protein-coding genes analyzed with RNA-Seq, a total of 3,729 (27%) were found to be DE in CHMs in comparison to normal first trimester placental tissues. Of these, 2,667 (72%) were up-regulated while 1,062 (28%) were down-regulated in CHM tissues (Supplementary Table S1, Figure 1). Of note, there were seven genes with placenta-specific expression among the top 20 transcripts most down-regulated but not up-regulated in CHMs (Table 3).

**Figure 2:**
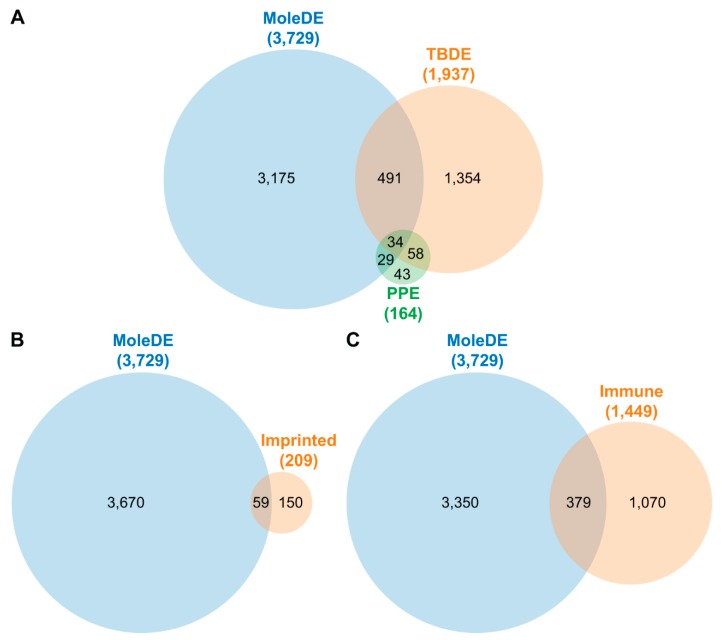
Venn diagrams of gene enrichment in complete hydatidiform moles. (**A**) Overlap between genes differentially expressed (DE) in complete hydatidiform moles (CHMs; MoleDE), and genes previously shown to have specific expression in the placenta (PPE) or during villous trophoblast differentiation (TBDE). (**B**) Overlap between genes DE in CHMs and previously described imprinted genes (Imprinted). (**C**) Overlap between genes DE in CHMs and genes previously shown to be involved in immune-related processes (Immune).

Enrichment analysis revealed significant enrichment (OR = 1.88, *p* = 0.0001) of placenta-specific genes (Supplementary Table S2, Figure 2A) among DE genes. Of interest, 50 out of 63 (79%) placenta-specific DE genes, found to be expressed mainly by the trophoblast, were down-regulated. Among functions of products of these genes were growth hormones (*CSHL1*, chorionic somatomammotropin hormone-like 1; *CSH1*, *CSH2)*, immune response (*LGALS14*, lectin, galactoside-binding, soluble, 14; *PSG4*), cell attachment (*EGFL6*, EGF-like domain multiple 6; *SMAGP*, small cell adhesion glycoprotein; *SVEP1*, Sushi, Von Willebrand factor type A, EGF and pentraxin domain containing protein 1), coagulation and blood pressure regulation (*AGTR1*, angiotensin II receptor type 1; *F13A1*, coagulation factor XIII A chain), and cell differentiation and developmental processes (*PAGE4*, PAGE family member 4; *PLAC1*, placenta specific 1; *RASA1*, RAS P21 protein activator 1).

Since majority of these placenta-specific genes are most highly expressed in the trophoblast, we wished to learn whether their differential expression may reflect alterations in developmental or functional processes of the trophoblast. Therefore, we examined whether genes involved in villous trophoblast differentiation [19] (Figure 2A) are enriched among molar DE genes. However, we found only minimal enrichment (OR = 1.15, *p* = 0.006).

We also analyzed 25 genes that are involved in epigenetic programming (four DNA methyltransferases, three methylcytosine dioxygenases, a cytidine deaminase, and 17 histone demethylases) and found two DNA methylation-related enzymes (*DNMT3A*, DNA methyltransferase 3 alpha; *TET3*, Tet methylcytosine dioxygenase 3) and four histone demethylases (*KDM4C*, lysine demethylase 4C; *KDM4D*; *KDM4E*; *KDM6B*) to be dysregulated in CHMs. *DNMT3A*, which was down-regulated, is required for genome-wide de novo DNA methylation and parental imprinting [20]. *TET3*, which was up-regulated, plays a key role in epigenetic reprogramming of the zygotic paternal DNA [21]. All four histone (lysine) demethylases [22] were up-regulated. In addition, there was an enrichment of genes impacted by parental imprinting (Supplementary Table S3, Figure 2B) among DE genes (OR = 2.38, *p* = 1.54 × 10^−6^).

Of note, the DE gene list contained 379 genes involved in immune-related functions (Supplementary Table S4, Figure 2C), of which 278 (73%) were overexpressed in CHMs. Genes contributing to this enrichment (OR = 1.82, *p* = 7.34 × 10^−18^) included those encoding several cytokines, chemokines and their receptors (*IL1A*, interleukin 1 alpha; *IL6*, *IL7*, *IL8/CXCL8*, *IL10*, *IL15*, *TGFB1*, transforming growth factor beta 1; *CXCR2*, C-X-C chemokine receptor type 2; *CXCR4*, *IL2RB*, *IL2RG*, *IL7R*, *IL15RA*, *TGFBR1*), integrins (e.g., *ITGA5*, integrin subunit alpha 5; *ITGAE*, *ITGAL*, *ITGAX*, *ITGB7*, integrin subunit beta 7), and galectins (*LGALS4*, *LGALS13*, *LGALS14*).

### 2.3. Dysregulated Biological Processes and Pathways in CHMs

Use of iPathwayGuide to investigate biological processes and pathways dysregulated in CHMs revealed that among 665 gene ontology (GO) biological processes, the most impacted were “cell adhesion”, “biological adhesion”, “multicellular organismal process”, and “signaling” (Supplementary Table S5). Applying Elim pruning that iteratively removes genes mapped to a significant GO term from more general higher level GO terms, identified “calcium ion binding”, “growth factor activity”, “extracellular matrix structural constituent”, and “G protein-coupled receptor activity” to be the most impacted among 150 dysregulated biological processes (Table 4, Supplementary Table S6).

We found the most impacted GO molecular functions to be “signaling receptor activity”, “molecular transducer activity”, and “gated channel activity” among 105 dysregulated molecular functions (Supplementary Table S7). Applying Elim pruning, “regulation of signaling receptor activity”, “cell adhesion”, “chemical synaptic transmission”, and “extracellular matrix organization” were identified as the most impacted among 628 dysregulated molecular functions (Table 5, Supplementary Table S8).

**Figure 3:**
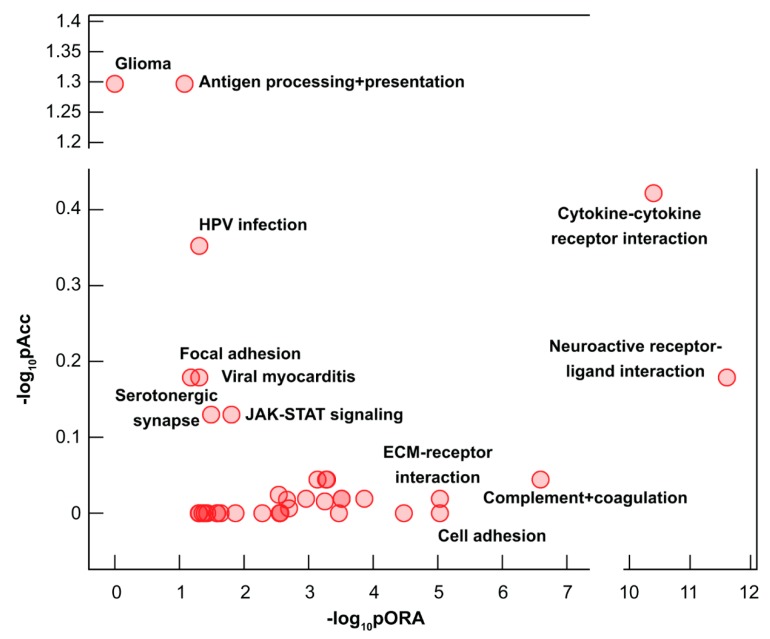
Pathways perturbation vs. over-representation in complete hydatidiform moles. Pathways are plotted according to two types of evidence computed by iPathwayGuide: over-representation on the x-axis (pORA) and the total pathway accumulation on the y-axis (pAcc). For both measures *p*-values are displayed on the negative log (base 10) scale. Extracellular matrix, ECM; human papilloma virus, HPV; Janus kinase, JAK; signal transducer and activator of transcription, STAT.

**Figure 4:**
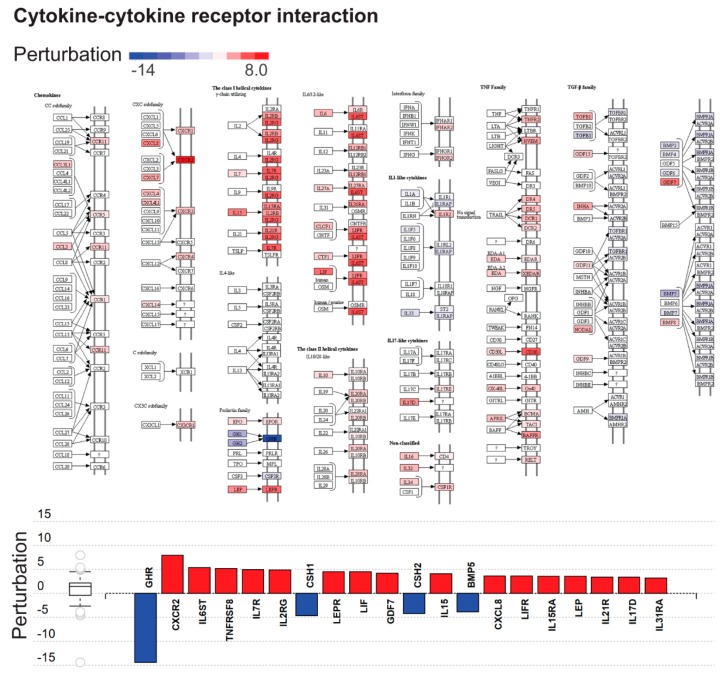
Cytokine–cytokine receptor interaction perturbation in complete hydatidiform moles. **Top**: The Kyoto Encyclopedia of Genes and Genomes (KEGG) pathway diagram (KEGG: 04060) is overlaid with the computed perturbation of each gene. Estimates of perturbation account for both for the genes′ measured fold change and for the accumulated perturbation propagated from any upstream genes (accumulation). The highest negative perturbation is shown in dark blue, and the highest positive perturbation in dark red. The legend describes values on the gradient. **Bottom**: Gene perturbation bar plot. All genes in the cytokine–cytokine receptor interaction pathway (KEGG: 04060) are ranked according to absolute perturbation values, negative values depicted in blue and positive values in red. The box and whisker plot on the left summarizes the distribution of all gene perturbations in this pathway, the box representing the first quartile, median and third quartile, while circles represent outliers.

The most impacted Kyoto Encyclopedia of Genes and Genomes (KEGG) pathways included “cytokine–cytokine receptor interaction”, “cell adhesion molecules”, “protein digestion and absorption”, and “neuroactive ligand–receptor interaction”, all important for placental functions (Table 6, Supplementary Table S9, Figure 3). An unanticipated finding was the most extensive dysregulation of “cytokine–cytokine receptor interaction” pathway (Figure 4) and “cell adhesion” pathway, both required for immune cell influx and activation. In addition, representation of 17 immune-related pathways among 38 dysregulated pathways (Table 6, Supplementary Table S9) reflects a strong immune component of molar pathogenesis.

### 2.4. Validation of RNA-Seq Results at the Protein Level

**Figure 5:**
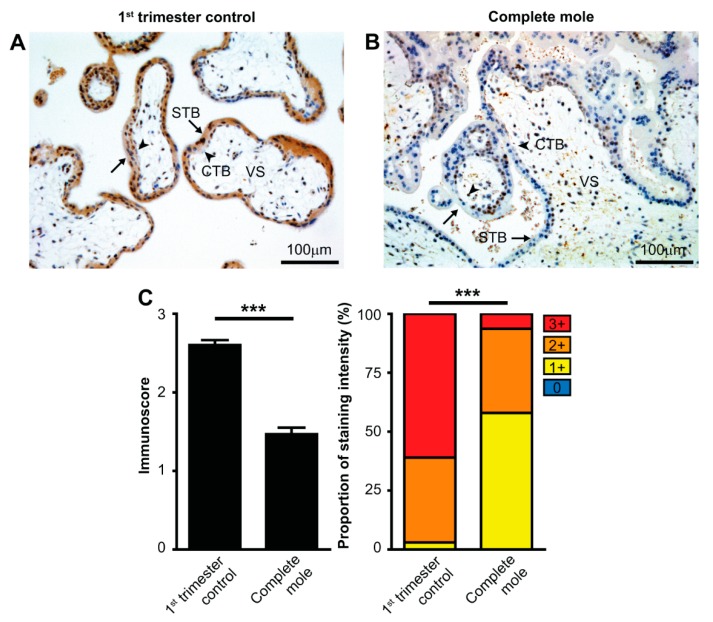
Differential expression of galectin-14 in syncytiotrophoblast in complete hydatidiform moles and first trimester control placentas. Five-µm-thick first trimester placental sections from normal pregnancy (**A**) or from CHMs (**B**) were stained for galectin-14 (gal-14). Chorionic villi exhibited intense syncytiotrophoblast cytoplasmic staining (arrows), while the villus stroma and cytotrophoblasts were negative (arrowheads) in normal placentas, and the syncytiotrophoblast layer had weak staining in CHMs. Representative images, hematoxylin counterstain, 200× magnifications. (**C**) Gal-14 immunoscores (mean ± SEM) and proportion of staining intensities in control placentas (*n* = 29) and CHMs (*n* = 23) are displayed on the left and right graphs, respectively. An unpaired t-test was used to compare mean immunoscores between control and CHM groups. Fisher’s exact test was used to test the difference in frequency of gal-14 immunostaining between the two groups. *** *p* < 0.001. Cytotrophoblast, CTB; syncytiotrophoblast, STB; villous stroma, VS.

First, we immunostained TMA slides for cyclin-dependent kinase inhibitor p57 (p57) expression to confirm the histopathology diagnosis of CHM at the molecular level. Out of 26 samples with the histopathology diagnosis of CHM, we detected cytotrophoblastic p57 staining in three samples, while 23 (88%) samples were devoid of p57 expression (Supplementary Figure S1), confirming the histopathological diagnosis of CHM in 23 samples. For validation of RNA-level findings, we conducted immunostaining of galectin-14 (gal-14), which is encoded by *LGALS14*, one of the genes most down-regulated in CHMs according to our RNA-Seq study. Average gal-14 immunoscores were 43% lower in CHMs than in gestational age-matched controls (1.47 ± 0.08 and 2.60 ± 0.06, respectively, *p* < 0.001). Additionally, the percentage of low-intensity staining (1+) was higher in molar than in control tissues (58% and 3%, respectively), while the percentage of high-intensity staining (3+) was lower in molar than in control tissues (6% and 61%, respectively), resulting in a significant difference in the distribution of gal-14 immunoscores (*p* < 0.001), consistent with RNA-Seq results for this locus (Figure 5).

## 3. Discussion

### 3.1. Principal Findings of This Study

High-dimensional transcriptomic analysis identified numerous distinctions between CHM and normal placenta, from which noteworthy patterns can be discerned. (1) the most highly expressed genes in first trimester normal placentas are those previously shown to have placenta-specific or predominantly placental expression; (2) in CHMs, overall gene expression levels are higher, while expression of placenta-specific transcripts are lower than in first trimester normal placentas; (3) the pathogenesis of CHMs involves the dysregulation of 27% of protein-coding genes expressed in both normal placentas and CHMs; (4) most DE genes (72%) in CHMs are up-regulated; (5) placental functions appear to be down-regulated in CHMs, since placenta-specific genes are enriched in DE genes, and most are down-regulated; (6) epigenetic reprogramming of the zygotic DNA and parental imprinting is dysregulated in CHMs; and (7) immune pathways are activated in CHMs as immune-related genes are enriched in DE genes and 17 immune-related pathways are impacted, mostly up-regulated, among 38 dysregulated pathways.

### 3.2. Placental Gene Expression and Functions are Down-Regulated in CHMs

In accord with previous studies [17,23], here we found genes with placenta-specific or predominant placental expression among the most highly expressed genes—12 out of 20—in first trimester normal placentas. These genes are involved in unique placental functions, including hormones, hormone synthesizing machinery, proteases, and immune proteins, which are pivotal for placental development, growth, signaling, and maternal–fetal immune tolerance. In CHMs, however, in spite of the overall median gene expression level being ~13% higher than in normal first trimester placentas, the overall median placenta-specific transcript level was 23% lower. This is also substantiated by the lower number of placenta-specific genes among the 20 most highly expressed transcripts in CHMs.

When investigating differential gene expression, we found 27% of the expressed protein-coding genes dysregulated in CHMs, of which 72% were up-regulated, underlining the generally higher gene expression levels in CHMs. Placenta-specific genes were enriched among DE genes, and there were much more down-regulated (79%) than up-regulated placenta-specific DE genes. In fact, the 20 most down-regulated genes included seven placenta-specific genes, while the 20 most up-regulated genes were devoid of these. Based on the functions of down-regulated placenta-specific genes, we can infer that placental growth and development, cell attachment, signaling, and immune defense are the most highly impacted processes in CHM. This was also supported by the classical pathway analysis, which revealed among the most impacted GO biological processes “cell adhesion”, “signaling”, “hormone activity”, “growth factor activity”, and “extracellular matrix structural constituent”, while the most impacted KEGG pathways included “cell adhesion molecules”, “neuroactive ligand–receptor interaction”, and “ECM–receptor interaction”.

### 3.3. Possible Causes of the Dysregulation of Placental Gene Expression in CHMs

Targeted gene expression studies on molar tissues and a recent meta-analysis of these studies showed that placenta-specific genes involved in villous trophoblast differentiation are also differentially expressed in molar tissues, and concluded that molar pathogenesis is, indeed, rooted in problems with trophoblast differentiation [16]. However, these studies were focusing on just a limited set of placenta-specific transcripts known to be differentially expressed during trophoblast differentiation, thus did not give a comprehensive insight into this important question. We recently discovered that abnormal villous trophoblast differentiation is at the root of the pathogenesis of early-onset preeclampsia, also reflected by profound placental dysregulation of placenta-specific genes [17,19]. Here we applied a similar non-targeted approach to investigate this issue. First, we intersected the list of DE genes in CHMs with the list of DE genes during villous trophoblast differentiation from our parallel study [19] and found only minimal enrichment for trophoblast differentiation genes in dysregulated genes in CHMs, much less than for placenta-specific genes. This suggests that trophoblast differentiation is moderately affected in CHMs, while the expression of placenta-specific genes is more extensively impacted. Among the affected placenta-specific genes we validated the down-regulation of *LGALS14* at the protein level. Since histopathological evidence shows the generally two-layered structure of villous trophoblasts in CHMs as in normal first trimester placentas, in spite of the histological and molecular evidence of locally more proliferative and or immortalized trophoblasts in CHMs [24,25,26,27,28,29,30,31], the histopathology of CHMs also underlines the generally moderate problem with villous trophoblast differentiation in CHMs.

A more compelling explanation would be that there is a problem with placental gene regulation due to the changes in DNA methylation and imprinting [32,33,34] since CHMs only contain paternal but not maternal genomes [35,36,37]. To test this hypothesis, we intersected the list of DE genes with the list of imprinted genes obtained from the GeneImprint database, and we found a significant enrichment of imprinted genes, underlining the link between CHM pathogenesis and affected paternal imprinting. In addition, we detected the down-regulation of the de novo DNA methyltransferase *DNMT3A* in CHMs, similar to the down-regulation of *DNMT3A* in absent fetal development, which is also the characteristics of *Dnmt3a* or *Dnmt3b* knockout mice [38]. Moreover, *TET3*, which is enriched specifically in the male pronucleus and responsible for paternal-genome conversion of 5mC into 5hmC [21], was two-fold up-regulated in CHMs. This is in good accordance with the paternal origin of all chromosomes in CHMs. Furthermore, four histone demethylases, which are active in the regulation of chromatin remodeling and gene expression and in influencing cellular differentiation, development, tumorigenesis, and inflammatory diseases [22,39], were found to be up-regulated in CHMs. These findings suggest that the epigenetic reprogramming of the zygotic DNA and parental imprinting are dysregulated in CHMs.

### 3.4. Immune Functions are Widely Dysregulated in CHMs

The pathogenesis of familial, biparental hydatidiform moles is caused by the inactivating mutations of *NLRP7* and *KHDC3L* [40,41,42], genes involved in imprinting and inflammation. Inactivating mutations in *NLRP7* inhibit cytokine (IL-1, interleukin-1; TNF, tumor necrosis factor) secretion by interfering with their trafficking, resulting in an in utero environment tolerogenic for the growth of these complete paternal allografts [41]. In this context, it is important that we found a wide dysregulation of immune pathways at the maternal–fetal interface in CHMs, which may also be at the heart of disease pathogenesis. To understand how this would be possible, we need to look first at immune processes in normal pregnancies. In healthy pregnant women, maternal–fetal immune tolerance mechanisms are complex and dynamic since embryo implantation in the first trimester is a pro-inflammatory process at the maternal–fetal interface, while the second trimester brings an anti-inflammatory milieu for the growing fetus in the womb, and the initiation of parturition at the end of pregnancy is characterized by a transition back towards a pro-inflammatory state [43,44,45,46]. These dynamic alterations in immune states are driven by the changing placental expression of molecules that regulate maternal immune responses [47,48,49,50,51,52,53,54,55,56,57,58]. Importantly, the dysregulated expression of immunoregulatory molecules at the maternal–fetal interface and the consequent disturbances in maternal–fetal immune regulation are strongly linked with alterations in the invasiveness of the trophoblast [59,60] and the development of severe pregnancy complications, such as miscarriages [61,62,63,64,65], preterm labor [66,67,68,69,70,71,72], or preeclampsia [73,74,75,76,77,78,79].

Herein, we detected the enrichment of genes involved in immune-related functions among DE genes in CHMs, and there were much more up-regulated than down-regulated immune-related genes. Of note, we noticed that *IL1A* and *IL33* were down-regulated while *FOXP3* (forkhead box P3), *IL7*, *IL10*, *LIF* (leukemia inhibitory factor), and *TGFB1* were up-regulated, which could theoretically induce a tolerogenic environment, similarly to familial hydatidiform moles. Indeed, previous studies described the infiltration of regulatory T cells, CD3+ T cells, and NK cells in CHMs at the implantation site [80,81,82]. This is in line with the finding herein on the up-regulation of chemokines and the activation of “cytokine–cytokine receptor interaction” and “cell adhesion molecules” pathways as well as biological processes such as “cell adhesion”, “biological adhesion”, and “signaling” needed for immune cell influx and immune responses.

On the other hand, we also detected the down-regulation of *LGALS13*, *LGALS14*, the up-regulation of various *HLA-I (A*, *B*, *C*, *E*, *F*, *G)* and *HLA-II (DMA*, *DMB*, *DPB1*, *DRA)* genes, complement pathway genes (e.g., *C1S*, *C3*, *C5*, *CR1*) and interleukins (e.g., *IL6*, *IL8/CXCL8*, *IL15*, *IL16*, *IL17D*), and the overall involvement of 17 immune-related pathways among 38 dysregulated pathways. Of note, eight out of 10 (e.g., *CD14*, *CD36*, *FCGRT*, neonatal Fc receptor; *LYVE1*, lymphatic vessel endothelial hyaluronan receptor 1) Hofbauer cell marker genes [83] were down-regulated in CHMs in our study in line with the relative lack of Hofbauer cells in CHM villi [84,85]. These findings suggest an enhanced maternal, over fetal, antigen-presenting and pro-inflammatory environment at the implantation site, which is also supported by the activation of the “antigen processing and presentation”, “phagosome”, “complement and coagulation cascades”, “allograft rejection”, and “graft-versus-host disease” pathways. This is in line with the fact that CHMs express only paternal antigens and thus represent complete allografts that could stimulate a vigorous alloimmune response from the maternal host [85], including local synthesis of complement C3 and complement activation [86]. Not only paternal alloantigens but also increased necrosis and apoptosis, due to the higher trophoblastic cell proliferation and turnover rate in CHMs [87,88], may trigger the production of several pro-inflammatory cytokines [89] and the activation of the complement system [89,90].

Based on all of these findings, we suggest that there is a complex immune dysregulation at the implantation site in CHMs, which include the parallel induction of anti- and pro-inflammatory processes due to the presentation of excess paternal antigens, aponecrotic trophoblastic debris, and the dysregulation of upstream transcription factors and other gene regulatory mechanisms. These are probably the consequences of abnormal paternal genome dosage and imprinting, resulting in impaired gene expression and placental development. To delineate the exact sequence of events, one would need to collect multiple samples from the implantation site in a time-series from patients and controls, and then to perform the extensive characterization of the localization and expression of RNA and protein signals. However, this type of sample collection is not possible due to ethical and technical reasons in spite of the fact that cellular and molecular characterization of such time-series materials would now be possible using, for example, RNA-Seq after laser capture microdissection or single-cell RNA-Seq as well as mass spectrometry-based tissue imaging. However, these techniques were not available for our current study.

### 3.5. Strengths and Limitations of the Study

According to our knowledge, this is the first non-hypothesis testing study that utilized various tools of high-dimensional biology to gain in-depth insights into the pathogenesis of CHMs. The strengths of the study are as follows: (1) strict clinical definitions and homogenous patient groups; (2) standardized, quick placental sample collection during surgeries and pregnancy terminations; (3) standardized histopathological examination of molar pregnancies and placentas based on international criteria; (4) high-quality sample processing and state-of-the-art high-dimensional methods for RNA and protein level analysis; (5) parallel expression profiling of various proteins on large tissue sets with tissue microarray and immunostaining followed by semi-quantitative immunoscoring and statistical analysis; and (6) the use of leading bioinformatics tools for RNA-Seq, gene ontology, and pathway analyses.

Limitations of the study are as follows: (1) the relatively modest number of cases due to the strict clinical and histopathological inclusion criteria used for patient enrollment. Paradoxically, this was also one of the most important strengths of our study; (2) data for cases and controls were retrieved from various sources in the RNA-Seq discovery study; (3) TMAs did not allow us the cellular compositional analysis of the investigated tissues due to the small core diameter size; and (4) it is possible that the differences in gene expression levels may originate from differential gene expression of particular cell types, differential cellular composition of CHMs and normal placentas, or the surgical/methodological differences in tissue sampling during elective terminations or CHM surgeries.

### 3.6. Concluding Remarks

In conclusion, our data shows that placental functions are down-regulated, expression of imprinted genes is altered, and immune pathways are activated in CHMs. Taken together, the results indicate that complex dysregulation of placental developmental and immune processes are pivotal for the pathogenesis of CHMs, providing new biological insight likely to inform molecular translational research addressing multiple stages of the natural history of CHM, such as assays for improved detection and refined diagnosis in early pregnancy [91], risk stratification with respect to malignant potential [92], and differential diagnosis of unexplained pregnancy loss [93].

## 4. Materials and Methods

### 4.1. Human Subjects, Clinical Samples, and Definitions

#### 4.1.1. Subjects in the RNA-Seq Discovery Study

All research participants completed a process of informed consent per the University of Southern California (USC) Health Sciences Institutional Review Board (protocols HS-09-00468 [12 November 2009) and HS-11-00095 (15 June 2011)]. Eligible subjects were identified upon presentation to Los Angeles County (LAC) and USC Medical Center. CHMs were identified clinically by a combination of ultrasonographic features (“snowstorm” or “cluster of grapes” appearance) with elevated blood hCG levels. Molar tissues were obtained between 8 2/7 and 14 0/7 weeks of gestational age by therapeutic surgical dilation and curettage. Confirmation of CHM diagnosis was determined by the histopathologic examination of surgical material. One subject who had CHM on the basis of clinical features was subsequently reclassified as having choriocarcinoma after pathological examination and was excluded from the study. Another subject with CHM was excluded due to pooling of several samples from various curettages into one sample, leaving the analyzed number of CHMs at four. Gestational age-matched control chorionic villous tissues were obtained at the Reproductive Options Clinic at LAC + USC Medical Center from surgical elective pregnancy terminations. To increase control group size, we also obtained RNA-Seq data of first trimester control chorionic villous tissues from a published study [23] (Table 7).

#### 4.1.2. Subjects in the Tissue Microarray Validation Study

GTD tissue samples had been collected during usual care of women who underwent treatment for GTD at the Department of Obstetrics and Gynecology, Keck School of Medicine, USC (Los Angeles, CA, USA). After approvals (protocols HS-14-00808 and HS-15-00720 for IRB approval, Lab agreement: 16-03-03) were obtained from the USC IRB, the institutional pathology database at the LAC Medical Center, Keck Medical Center of USC, and Norris Comprehensive Cancer Center was utilized to identify GTD cases by searching for the keywords “gestational trophoblastic disease”, “molar pregnancy”, “hydatidiform mole”, “complete mole”, and “invasive mole” between 1 January 2000 and 11 September 2017. Inclusion criteria included GTD cases from whom archived histopathology specimens were available. GTD cases without salient clinical information were excluded.

For eligible GTD cases, data describing clinical and demographic features, tumor characteristics and markers, treatment course, and survival outcomes were abstracted from the medical record. Clinical and demographic features included patient age at diagnosis of GTD, ethnicity, pregnancy history including interval months from the last menstrual period of index pregnancy, body mass index, and medico-surgical history. Tumor characteristics and markers included the World Health Organization (WHO) score, histology subtype, tumor size, pretreatment beta-hCG, metastatic sites, and number. Treatment course included surgical intervention (dilation and curettage, or hysterectomy) and chemotherapy (type, cycle number, and response). Survival outcomes included progression-free survival (PFS) and overall survival. Progression-free survival was determined as the time interval between treatment initiation for GTD and the date of first recurrence or last follow-up date if there was no recurrence. Overall survival was determined as the time interval between treatment initiation for GTD and the date of death related to GTD or last follow-up date if the patient was alive.

Of 311 cases that were initially identified, 44 CHM cases were chosen for TMA based on tissue availability (Table 8 and Table 9). We placed no restriction on the age of gestation and found pregnancies to be dated according to ultrasound scans between 5 and 15 weeks. Patients with multiple pregnancies were excluded, and specimens and data were de-identified for use in research according to procedures approved by the Institutional Review Boards of USC. From the 44 GTD tissues identified between 4 2/7 and 36 1/7 weeks of gestational age, the TMA project included 26 samples obtained from therapeutic surgical dilation and curettage within the first trimester (13 6/7 weeks) and having good tissue quality. The 26 samples were obtained from 23 patients. One patient provided two specimens at the same sampling time, while another patient provided three specimens at different time points.

Control samples of first trimester placental tissue (*n* = 29) matched by gestational age to GTD samples were collected prospectively at the Maternity Private Clinic (Budapest, Hungary) and deposited into the Perinatal Biobank of the Research Centre for Natural Sciences, Hungarian Academy of Sciences (Budapest, Hungary). Informed consent for use of the material in research was obtained from women prior to sample collection, and specimens and data were stored anonymously. The research was approved by the Health Science Board of Hungary (TUKEB 4834-0/2011-1018EKU), and all use of tissue and data in this research conformed to the principles set out in the World Medical Association (WMA) Declaration of Helsinki. Placentas were collected from pregnancies voluntarily terminated by surgical dilation and curettage between 5 and 14 weeks of gestation according to ultrasound scans, excluding multiple pregnancies (Table 8).

### 4.2. Experimental Procedures

#### 4.2.1. Sample Collection and Preparation for RNA-Seq

Tissue samples were rinsed with sterile saline and examined under a lightbox for content confirmation and selection of molar tissue or chorionic villi. Samples were then stored in RNA*later* RNA stabilization reagent (approximately 10 μL reagent per 1mg of tissue, Qiagen, Germantown, MD, USA) at 37 °C for a maximum of 24 h until collected for dissection. Samples were then dissected into ~1 g tissue aliquots and stored at −80 °C. Stored samples were then thawed at room temperature and homogenized with electric homogenizer. Total RNA was isolated using the protocol provided with the Qiagen RNeasy Mini Kit. RNA quantity was measured by a Nanodrop ND-8000 analyzer (Thermo Scientific, Waltham, MA, USA). Samples were then stored at −80 °C until used.

#### 4.2.2. RNA Sequencing

Library preparation and paired-end sequencing were performed by the USC Epigenome Core Laboratory. Double-stranded cDNA fragments were synthesized from mRNA, ligated with adapters, and size-selected for library construction according to Illumina protocol [94]. RNA sequencing was carried out on Illumina Genome Analyzer (Illumina, San Diego, CA, USA).

#### 4.2.3. Histopathological Examinations

All slides cut from formalin-fixed, paraffin-embedded (FFPE) molar tissue blocks were retrieved from our USC + LAC histology archives. The slides were reviewed for confirmation of the diagnosis and regions were identified for TMA construction. The blocks from the matching slides were retrieved and submitted for TMA construction.

Samples of tissue from first trimester placentas had been fixed in 10% neutral-buffered formalin and then embedded in paraffin. Five µm sections were cut from tissue blocks, stained with hematoxylin and eosin (H&E), and examined using light microscopy. A perinatal pathologist blinded to patients’ clinical information, except for the gestational age, examined the histopathology of placental samples using standard perinatal pathological protocol and previously-published diagnostic criteria [95,96,97], and identified regions of each tissue block from which to sample cores.

#### 4.2.4. Tissue Microarray Construction

A material transfer agreement between the Hungarian Academy of Sciences and USC (No: 1996/2017) enabled the construction of TMAs containing tissues collected at USC and at Maternity Private Clinic and deposited into the Perinatal Biobank. Four TMAs were constructed, each containing three cylindrical cores two mm in diameter from each sample of first trimester FFPE placental (*n* = 29) or GTD (*n* = 26) tissue. Cores from the same sample were transferred into recipient paraffin blocks adjacent to each other using an automated tissue arrayer (TMA Master II, 3DHISTECH, Budapest, Hungary). Each paraffin block contained three cores from all tissues, with liver included as negative control and third trimester placenta as positive control material.

#### 4.2.5. Immunohistochemistry and Immunoscoring

To validate the histological diagnosis of CHM, we conducted immunostaining for p57, which is expressed from a paternally imprinted gene and is a recognized diagnostic marker in CHM [12,98,99]. To validate RNA level findings at the protein level for one down-regulated gene, five-µm-thick sections were cut from each TMA onto adhesive glass slides (SuperFrost Ultra Plus, TS Labor, Budapest, Hungary), and immunostained for p57 and galectin-14 using antibodies and conditions listed in Table 10. Briefly, sections were dewaxed using xylene and rehydrated in graded alcohol series.

For p57 immunostaining, endogen peroxidases were blocked using 10% H_2_O_2_ in methanol for 20 min, then antigen retrieval was performed in Tris-EDTA pH 9.0 buffer for 20 min at 96 °C. For galectin-14 immunostaining, endogen peroxidases were blocked using 1% H_2_O_2_ in methanol for 20 min, then antigen retrieval was performed in Tris-EDTA pH 9.0 buffer for 32 min at 100 °C. In all staining procedure, after 10 min Novolink protein blocking (Leica-Novocastra, Wetzlar, Germany), the sections were incubated at room temperature with antibodies and then with reagents of the Novolink Polymer Detection System (Leica-Novocastra). Between incubation steps, the sections were washed trice for 5 min in Tris-buffered saline (pH 7.4). Finally, sections were counterstained with hematoxylin, and these were mounted (DPX Mountant; Sigma-Aldrich, St. Louis, MO, USA) after dehydration.

Immunostained TMA images were semi-quantitatively scored by two examiners blinded to the clinical information with an immunoreactive score modified from one previously used in our studies [18,65,74,100,101,102]. Trophoblastic immunostaining intensity was graded as 0 = negative, 1 = weak, 2 = intermediate, and 3 = strong. For each specimen, all villi and trophoblastic tissues in a random field of each of the cores were evaluated by both examiners using Panoramic Viewer 1.15.4 (3DHISTECH Ltd., Budapest, Hungary).

### 4.3. Data Analysis

#### 4.3.1. Analysis of mRNA Expression (RNA-Seq Data)

For data produced from five samples, FASTQ files were generated by Illumina’s pipeline and read quality was assessed by FastQC (http://www.bioinformatics.babraham.ac.uk/projects/fastqc/). Subsequently, reads were submitted to alignment with HISAT2 (v2.1.0) [103]. The mapping was made using default parameters with reference human genome GRCh38 (downloaded from Illumina iGenomes). Aligned BAM files were indexed and sorted with Samtools (v0.1.18) [104] for downstream analysis. Genomic features and read count matrices were obtained using featureCounts (v1.5.2) [105] based on annotation file hg38 (RefSeq track of UCSC Table Browser).

RNA-Seq count data for additional late first trimester control placentas (22 XY and 17 XX) were downloaded from GEO (AccNo: GSE109082) [23]. Differential gene expression analysis was performed using the R package DESeq2 [106,107].

Because data included differing amounts of non-coding RNA leading to high numbers of DE genes presumed to be spurious, we restricted the differential gene expression analysis to 19,690 protein-coding genes based on the ENSEMBL “biotype” annotation. We further excluded genes with zero read count in any sample, resulting in a final analytic set of data on 14,022 genes. As a set of DE transcripts, we selected those with a fold change of ≥2 between CHM and control, while holding the false discovery rate (pFDR) to <0.05.

To estimate absolute levels of expression, we normalized RNA-Seq count data by the geometrical mean of measured counts of four housekeeping genes (*TBP*, TATA-box binding protein; *CYC1*, cytochrome C1; *UBC*, ubiquitin C; *TOP1*, topoisomerase (DNA) I), and calculated the mean expression both in CHM and control groups of samples.

#### 4.3.2. Pathway Analysis

To identify significantly impacted pathways, biological processes, and molecular functions, we compared expression between CHM and control groups using Advaita Bio’s iPathwayGuide (https://www.advaitabio.com/ipathwayguide). This software analysis tool implements the “impact analysis” approach that takes into consideration the direction and type of all signals on a pathway and the position, role and type of every gene, as described in [108].

#### 4.3.3. Enrichment Analyses

We used Fisher’s exact test to test enrichment of the DE transcript set with genes previously demonstrated to have expression patterns of particular relevance: (1) genes with placenta-specific expression (*n* = 164, Supplementary Table S2) [17]; (2) genes involved in villous trophoblast differentiation (*n* = 1,937; GEO: GSE130339, the list was provided by authors of an unpublished work [19]); (3) genes imprinted in humans (*n* = 209; imprinted gene databases, www.geneimprint.com, Supplementary Table S3 and 4 genes related to immune processes (*n* = 1,449; www.innatedb.com; the final list is the manual curation and combination of Immport, Immunogenetic Related Information Source, and Immunome Database Gene Lists, Supplementary Table S4).

#### 4.3.4. Analysis of TMA Data

For each core, immunostain was scored by each of two examiners using a scale of 0 to 3, 0 representing none and 3 strong immunostaining. Quantitative immunoscores were determined as follows. For each core, immunoscores from examiners were first averaged to represent that core, and resulting values for each of the three cores representing one sample were averaged to represent that sample. Mean immunoscores between control and CHM groups were compared using the unpaired t-test. The Fisher’s exact test was performed to test the distribution of gal-14 immunoscores between the control and CHM groups. Results were considered statistically significant at * *p* < 0.05, ** *p* < 0.01, and *** *p* < 0.001.

#### 4.3.5. Data Availability

USC RNA-Seq data was deposited to the Gene Expression Omnibus (GEO) database according to the MIAME guidelines (accession number: GSE138250).

## Figures and Tables

**Table 1:** Genes encoding the 20 transcripts most highly expressed in first trimester placentas.

Gene Symbol	Entrez ID	Base Mean	lfcSE	Log_2_ FC	pFDR	*p*-Value	Control Mean
*CGA*	1081	164,079.45	0.63	0.07	0.93	0.91	155,778.30
***FN1***	**2335**	**141,988.22**	**0.56**	**0.32**	**0.64**	**0.57**	**135,448.78**
*TFPI2*	7980	125,046.51	0.58	−1.16	0.08	0.05	127,071.15
***CSH1***	**1442**	**87,798.80**	**0.67**	**−4.58**	**0.00**	**0.00**	**93,037.03**
*EEF1A1*	1915	84,023.14	0.15	−1.20	0.00	0.00	87,193.18
*PEG10*	23089	77,282.40	0.24	−1.74	0.00	0.00	82,299.13
***CSH2***	**1443**	**61,726.09**	**0.81**	**−4.23**	**0.00**	**0.00**	**65,433.13**
*COL3A1*	1281	60,159.32	0.29	−1.30	0.00	0.00	63,035.40
***KISS1***	**3814**	**65,121.70**	**0.65**	**0.13**	**0.88**	**0.84**	**61,457.48**
***ADAM12***	**8038**	**44,435.52**	**0.35**	**−0.06**	**0.90**	**0.86**	**43,427.10**
***CYP19A1***	**1588**	**40,376.35**	**0.53**	**−1.27**	**0.03**	**0.02**	**41,478.58**
*SPP1*	6696	37,734.38	0.41	−4.02	0.00	0.00	40,796.23
***TGM2***	**7052**	**39,725.43**	**0.36**	**−0.36**	**0.39**	**0.31**	**39,553.03**
***PSG3***	**5671**	**38,487.88**	**0.51**	**−1.05**	**0.07**	**0.04**	**39,153.23**
***PAPPA***	**5069**	**35,537.55**	**0.59**	**−1.35**	**0.04**	**0.02**	**36,733.78**
***PSG4***	**5672**	**33,870.64**	**0.59**	**−3.17**	**0.00**	**0.00**	**36,069.85**
*COL4A1*	1282	36,995.51	0.37	0.33	0.46	0.37	35,055.03
***PAPPA2***	**60676**	**34,031.77**	**0.48**	**0.31**	**0.60**	**0.52**	**32,044.98**
***FBN2***	**2201**	**30,126.49**	**0.26**	**−1.57**	**0.00**	**0.00**	**31,930.00**
*ACTB*	60	31,472.69	0.22	0.39	0.11	0.07	29,870.08

Placenta-specific genes are shown in bold blue. False discovery rate, pFDR; fold change, FC; log fold change standard error, lfcSE.

**Table 2:** Genes encoding the 20 transcripts most highly expressed in complete hydatidiform moles.

Gene Symbol	Entrez ID	Base Mean	lfcSE	Log_2_ FC	pFDR	*p*-Value	Control Mean	CHM Mean
HBB *	3043	33,412.13	0.49	7.74	0.00	0.00	1,608.00	373,335.00
*CGA*	1081	164,079.45	0.63	0.07	0.93	0.91	155,778.30	189,408.50
***FN1***	**2335**	**141,988.22**	**0.56**	**0.32**	**0.64**	**0.57**	**135,448.78**	**184,274.75**
PAEP *	5047	7,444.75	0.96	7.11	0.00	0.00	545.88	95,119.25
***KISS1***	**3814**	**65,121.70**	**0.65**	**0.13**	**0.88**	**0.84**	**61,457.48**	**78,481.00**
GDF15 *	**9518**	**24,551.03**	**0.58**	**1.70**	**0.01**	**0.00**	**19,462.58**	**73,765.25**
*TFPI2*	7980	125,046.51	0.58	−1.16	0.08	0.05	127,071.15	66,657.75
IGF2 *	3481	26,174.25	0.22	1.20	0.00	0.00	23,075.75	60,786.25
HBA2 *	3040	5,986.27	0.56	4.95	0.00	0.00	1,590.28	52,014.25
*COL4A1*	1282	36,995.51	0.37	0.33	0.46	0.37	35,055.03	49,353.25
***ADAM12***	**8038**	**44,435.52**	**0.35**	**−0.06**	**0.90**	**0.86**	**43,427.10**	**47,781.00**
LEP *	**3952**	**6,372.49**	**0.65**	**3.52**	**0.00**	**0.00**	**3,092.85**	**45,604.50**
*ACTB*	60	31,472.69	0.22	0.39	0.11	0.07	29,870.08	45,004.50
***PAPPA2***	**60676**	**34,031.77**	**0.48**	**0.31**	**0.60**	**0.52**	**32,044.98**	**44,734.75**
EEF1A1 *	1915	84,023.14	0.15	−1.20	0.00	0.00	87,193.18	43,702.00
*AHNAK*	79026	25,150.93	0.24	0.66	0.01	0.01	23,452.63	43,214.25
***CGB5***	**93659**	**18,949.99**	**0.70**	**1.13**	**0.16**	**0.11**	**16,161.30**	**40,758.75**
*FLT1*	2321	19,476.41	0.32	0.96	0.01	0.00	17,411.10	38,701.00
FSTL3 *	10272	7,919.03	0.63	2.57	0.00	0.00	5,172.10	36,659.00
CGB3 *	**1082**	**13,840.61**	**0.66**	**1.49**	**0.04**	**0.02**	**11,254.20**	**35,670.25**

Placenta-specific genes are shown in bold blue. Differentially expressed genes are shown with asterisks. Complete hydatidiform mole, CHM; false discovery rate, pFDR; fold change, FC; log fold change standard error, lfcSE.

**Table 3:** Genes encoding the 20-20 transcripts with highest and lowest expression in complete hydatidiform moles.

Gene Symbol	Entrez ID	Base Mean	Log_2_ FC	lfcSE	*p*-Value	pFDR
*HBB*	3043	33,412.13	7.74	0.49	4.64 × 10^−57^	4.06 × 10^−54^
*PAEP*	5047	7,444.75	7.11	0.96	1.70 × 10^−13^	2.45 × 10^−12^
*CP*	1356	307.65	7.01	0.69	1.38 × 10^−24^	8.39 × 10^−23^
*C2CD4B*	388125	59.15	6.66	0.54	6.42 × 10^−35^	1.25 × 10^−32^
*IRX3*	79191	99.72	6.32	0.53	1.90 × 10^−32^	2.77 × 10^−30^
*BEST1*	7439	801.46	6.23	0.76	2.40 × 10^−16^	5.15 × 10^−15^
*DSEL*	92126	88.53	6.12	0.25	4.36 × 10^−128^	3.05 × 10^−124^
*B4GALNT3*	283358	83.11	5.85	0.51	2.80 × 10^−30^	3.32 × 10^−28^
*PGR*	5241	62.02	5.85	0.49	3.04 × 10^−33^	4.90 × 10^−31^
*ZNF623*	9831	59.42	5.77	0.18	1.30 × 10^−214^	1.82 × 10^−210^
*KIAA1324*	57535	79.48	5.58	0.46	1.77 × 10^−33^	3.03 × 10^−31^
*IGFBP7*	3490	658.18	5.52	0.46	3.03 × 10^−33^	4.90 × 10^−31^
*RORC*	6097	36.06	5.47	0.43	3.04 × 10^−36^	6.86 × 10^−34^
*DEFB1*	1672	47.01	5.44	0.58	5.59 × 10^−21^	2.16 × 10^−19^
*PKHD1*	5314	74.85	5.36	0.71	5.31 × 10^−14^	8.23 × 10^−13^
*ADCY1*	107	53.12	5.33	0.37	6.31 × 10^−48^	2.95 × 10^−45^
*MAPT*	4137	23.64	5.30	0.48	4.39 × 10^−28^	4.15 × 10^−26^
*PRUNE2*	158471	140.93	5.29	0.49	9.85 × 10^−27^	7.89 × 10^−25^
*GALNT15*	117248	23.91	5.18	0.47	8.01 × 10^−28^	7.28 × 10^−26^
*WDR38*	401551	74.42	5.12	1.02	5.62 × 10^−07^	2.74 × 10^−06^
*HAPLN1*	1404	3,248.02	−3.66	0.31	1.18 × 10^−32^	1.80 × 10^−30^
*CD36*	948	2,906.67	−3.71	0.39	6.13 × 10^−22^	2.71 × 10^−20^
*WNT2*	7472	1,855.95	−3.72	0.33	8.29 × 10^−29^	8.30 × 10^−27^
***EGFL6***	**25975**	**4,141.42**	**−3.75**	**0.29**	**1.50 × 10^−37^**	**3.69 × 10^−35^**
*BMP5*	653	1,150.58	−3.86	0.37	3.40 × 10^−25^	2.29 × 10^−23^
***F13A1***	**2162**	**7,493.03**	**−3.98**	**0.41**	**1.75 × 10^−22^**	**8.18 × 10^−21^**
*SPP1*	6696	37,734.38	−4.02	0.41	6.04 × 10^−23^	2.97 × 10^−21^
*HBG2*	3048	4,458.52	−4.05	0.38	4.57 × 10^−26^	3.35 × 10^−24^
*MFSD14A*	64645	776.11	−4.19	0.26	5.42 × 10^−59^	5.96 × 10^−56^
***CSH2***	**1443**	**61,726.09**	**−4.23**	**0.81**	**1.94 × 10^−07^**	**1.03 × 10^−06^**
***AGTR1***	**185**	**664.42**	**−4.27**	**0.38**	**2.85 × 10^−29^**	**3.03 × 10^−27^**
*COX4I2*	84701	131.49	−4.28	0.47	1.36 × 10^−19^	4.47 × 10^−18^
***SVEP1***	**79987**	**15,505.08**	**−4.33**	**0.46**	**5.20 × 10^−21^**	**2.01 × 10^−19^**
*LYVE1*	10894	2,663.78	−4.56	0.34	6.68 × 10^−42^	2.23 × 10^−39^
***CSH1***	**1442**	**87,798.80**	**−4.58**	**0.67**	**5.61 × 10^−12^**	**6.40 × 10^−11^**
*AREG*	374	710.42	−4.64	0.45	4.88 × 10^−25^	3.19 × 10^−23^
*SPESP1*	246777	321.55	−5.11	0.40	1.75 × 10^−36^	4.01 × 10^−34^
*RPS10*	6204	742.05	−5.28	0.44	3.32 × 10^−33^	5.29 × 10^−31^
***CSHL1***	**1444**	**2,846.50**	**−5.43**	**0.41**	**2.25 × 10^−40^**	**6.85 × 10^−38^**
*ZC3H11A*	9877	2,887.90	−5.95	0.32	4.7 × 10^−76^	8.29 × 10^−73^

Placenta-specific genes are depicted with bold blue. False discovery rate, pFDR; fold change, FC; log fold change standard error, lfcSE.

**Table 4:** Twenty most impacted Gene Ontology biological processes in complete hydatidiform moles.

GO ID	GO Name	Count DE	Count All	p Elim Pruning
GO:0005509	calcium ion binding	178	426	1.60 × 10^−13^
GO:0008083	growth factor activity	50	84	5.50 × 10^−11^
GO:0005201	extracellular matrix structural constituent	74	130	3.00 × 10^−08^
GO:0004930	G protein-coupled receptor activity	90	188	9.00 × 10^−07^
GO:0008201	heparin binding	47	99	2.40 × 10^−06^
GO:0005125	cytokine activity	36	70	3.60 × 10^−06^
GO:0030020	extracellular matrix structural constituent conferring tensile strength	22	35	4.00 × 10^−06^
GO:0004888	transmembrane signaling receptor activity	192	421	4.80 × 10^−06^
GO:0042605	peptide antigen binding	13	17	1.70 × 10^−05^
GO:0039706	co-receptor binding	8	8	1.90 × 10^−05^
GO:0005178	integrin binding	42	91	2.00 × 10^−05^
GO:0005044	scavenger receptor activity	24	43	2.60 × 10^−05^
GO:0038023	signaling receptor activity	259	568	3.10 × 10^−05^
GO:0005249	voltage-gated potassium channel activity	23	27	3.70 × 10^−05^
GO:0004252	serine-type endopeptidase activity	30	60	4.60 × 10^−05^
GO:0005179	hormone activity	19	34	1.80 × 10^−04^
GO:0008331	high voltage-gated calcium channel activity	6	6	2.90 × 10^−04^
GO:0020037	heme binding	29	63	3.90 × 10^−04^
GO:0030506	ankyrin binding	10	14	4.40 × 10^−04^
GO:0003996	acyl-CoA ligase activity	7	8	4.40 × 10^−04^

Differentially expressed, DE; Gene Ontology, GO.

**Table 5:** Twenty most impacted Gene Ontology molecular functions in complete hydatidiform moles.

GO ID	GO Name	Count DE	Count All	p Elim Pruning
GO:0010469	regulation of signaling receptor activity	143	285	3.90 × 10^−14^
GO:0007155	cell adhesion	407	1,014	2.00 × 10^−13^
GO:0007268	chemical synaptic transmission	165	397	3.60 × 10^−07^
GO:0030198	extracellular matrix organization	122	271	3.80 × 10^−07^
GO:0030449	regulation of complement activation	22	33	1.20 × 10^−06^
GO:0006958	complement activation, classical pathway	16	22	6.40 × 10^−06^
GO:0034765	regulation of ion transmembrane transport	130	295	1.50 × 10^−05^
GO:0045669	positive regulation of osteoblast differentiation	29	54	1.50 × 10^−05^
GO:0007156	homophilic cell adhesion via plasma membrane adhesion molecules	36	76	5.40 × 10^−05^
GO:0007186	G protein-coupled receptor signaling pathway	196	516	6.00 × 10^−05^
GO:0007166	cell surface receptor signaling pathway	686	2,116	8.60 × 10^−05^
GO:0007601	visual perception	51	123	1.50 × 10^−04^
GO:0006957	complement activation, alternative pathway	8	9	1.50 × 10^−04^
GO:0006805	xenobiotic metabolic process	32	68	1.60 × 10^−04^
GO:0002576	platelet degranulation	44	103	1.80 × 10^−04^
GO:0042102	positive regulation of T cell proliferation	32	60	2.20 × 10^−04^
GO:0035115	embryonic forelimb morphogenesis	17	29	2.20 × 10^−04^
GO:0030501	positive regulation of bone mineralization	19	34	2.20 × 10^−04^
GO:0048662	negative regulation of smooth muscle cell proliferation	23	39	2.30 × 10^−04^
GO:0035116	embryonic hindlimb morphogenesis	14	22	2.20 × 10^−04^

Differentially expressed, DE; Gene Ontology, GO.

**Table 6:** Top 20 most impacted pathways in complete hydatidiform moles.

Pathway Name	*p*-value	pFDR
**Cytokine-cytokine receptor interaction**	**1.38 × 10^−07^**	**2.31 × 10^−05^**
Cell adhesion molecules (CAMs)	1.43 × 10^−07^	2.31 × 10^−05^
Protein digestion and absorption	6.20 × 10^−07^	6.68 × 10^−05^
Neuroactive ligand-receptor interaction	1.32 × 10^−06^	1.07 × 10^−04^
**Complement and coagulation cascades**	**6.91 × 10^−06^**	**4.46 × 10^−04^**
Hypertrophic cardiomyopathy (HCM)	1.05 × 10^−05^	4.87 × 10^−04^
Extracellular matrix (ECM)-receptor interaction	1.06 × 10^−05^	4.87 × 10^−04^
**Autoimmune thyroid disease**	**2.91 × 10^−05^**	**1.17 × 10^−03^**
**Allograft rejection**	**7.36 × 10^−05^**	**2.39 × 10^−03^**
**Graft-versus-host disease**	**8.01 × 10^−05^**	**2.39 × 10^−03^**
**Antigen processing and presentation**	**8.15 × 10^−05^**	**2.39 × 10^−03^**
**Intestinal immune network for IgA production**	**9.94 × 10^−05^**	**2.68 × 10^−03^**
**Staphylococcus aureus infection**	**1.18 × 10^−04^**	**2.94 × 10^−03^**
Metabolism of xenobiotics by cytochrome P450	1.64 × 10^−04^	3.63 × 10^−03^
**Hematopoietic cell lineage**	**1.73 × 10^−04^**	**3.63 × 10^−03^**

Immune-related pathways are shown in bold blue. False discovery rate, pFDR.

**Table 7:** Demographic characteristics of patients in the RNA-Seq discovery study.

	Patients Providing GTD Tissue Samples (n = 4)	Patients Providing Placenta Samples (n = 40)
Race/ethnicity Hispanic Caucasian Asian African-American Unknown/other	2 - 1 - 1	6 34 - - -
Age at procedure	25.7	39.2
Histologic diagnosis Complete mole Incomplete mole Invasive mole	3 - 1	- - -
Sex chromosome content XY XX	- 4	22 18
Gestational age at tissue collection (days)	79.2 (58–98)	84.2 (77–124)

Gestational trophoblastic disease, GTD.

**Table 8:** Demographic characteristics of patients in the TMA validation study.

	Patients Providing GTD Tissue Samples (n = 23 *)	Patients Providing Placenta Samples (n = 29)
Race/ethnicity ^a^ Hispanic Non-Hispanic white Asian African-American Unknown/other	18 (78%) 2 (9%) 0 0 3 (13%)	

		29 (100%)



Age (years) at procedure ^b^	31.9 ± 9.5	30.4 ± 6.4
Comorbidities Hypertension ^a^ Diabetes ^a^ beta-hCG (mIU/mL) ^c^	1 (4%) 1 (4%) 185,277 (70,349–387,812)	
Histologic diagnosis ^a^		
Complete mole	26 (100%)	0 (0%)
p57 immunostaining confirmation of CHM	23 (88%)	
Gestational age at the procedure (days) ^b^	64.4 ± 18.2	62.3 ± 11.8

* A total of 26 samples altogether; one case with two specimens at the same time; another case with three specimens at different time points. ^a^ Data are presented as number (percentage). ^b^ Data are presented as mean ± SD. ^c^ Data are presented as median (IQR). Cyclin-dependent kinase inhibitor p57; human chorionic gonadotropin, hCG; gestational trophoblastic disease, GTD; tissue microarray, TMA.

**Table 9:** Criteria for categorizing gestational trophoblastic disease cases and number of samples in each category included in the tissue microarray study.

Grouping	Definition	Sample No. *
1	D&C then cured	15
2a	D&C then persistent GTD, cured by first-line single agent	1
2b	D&C then persistent GTD, cured by upfront hysterectomy	4
2c	D&C then persistent GTD, cured by upfront hysterectomy and adjuvant chemotherapy	0
3a	D&C then persistent GTD, received first-line single agent but failed	3
3b	D&C then persistent GTD, received first-line multi-agent and cured	0
3c	D&C then persistent GTD, received first-line multi-agent and failed	0
3d	D&C then persistent GTD, received upfront hysterectomy but recurred	0
4	Recurrent GTD	3

* A total of 26 samples altogether; one case with two specimens at the same time; another case with three specimens at different time points. Dilatation and curettage, D&C; gestational trophoblastic disease, GTD.

**Table 10:** Immunohistochemistry conditions.

Primary Antibody (Concentration/Dilution, Distributor)	Secondary Antibody (Dilution, Distributor)	Detection Antibody (Distributor)	Detection System (Distributor)
monoclonal mouse anti-human p57 antibody (1:3000) (code: MA5-11309, Thermo Fisher Scientific, Waltham, MA, USA)	-	Novolink detections system (Leica-Novocastra, Wetzlar, Germany)	Novolink DAB/substrate kit (Leica-Novocastra, Wetzlar, Germany)
recombinant, His_6_-tagged anti-human gal-14 antibody (0.65 µg/mL) [provided by Prof. R. Romero (PRB, NIH)]	monoclonal mouse anti-His_6_-tag antibody (1:200) (code: 05-959, Merck-Millipore, Burlington, MA, USA)		

## Supplementary Materials

Supplementary materials can be found at https://www.mdpi.com/1422-0067/20/20/4999/s1.

## Abbreviations

CHM	complete hydatidiform mole
ECM	extracellular matrix
FFPE	formalin-fixed paraffin-embedded
gal-14	galectin-14
GO	Gene Ontology
GTD	gestational trophoblastic disease
GTN	gestational trophoblastic neoplasia
hCG	human chorionic gonadotropin
IL	interleukin
KEGG	Kyoto Encyclopedia of Genes and Genomes
p57	cyclin-dependent kinase inhibitor p57
pFDR	false discovery rate
TMA	tissue microarray
