# Supplementary material for: Dysregulation of Placental Functions and Immune Pathways in Complete Hydatidiform Moles
Source: Int J Mol Sci. 2019 Oct 10;20(20):4999. doi: 10.3390/ijms20204999 (PMC6829352; doi:10.3390/ijms20204999)
Supplement: Supplementary file 1 [file ijms-20-04999-s001.zip › Supplementary Figure 1.docx]

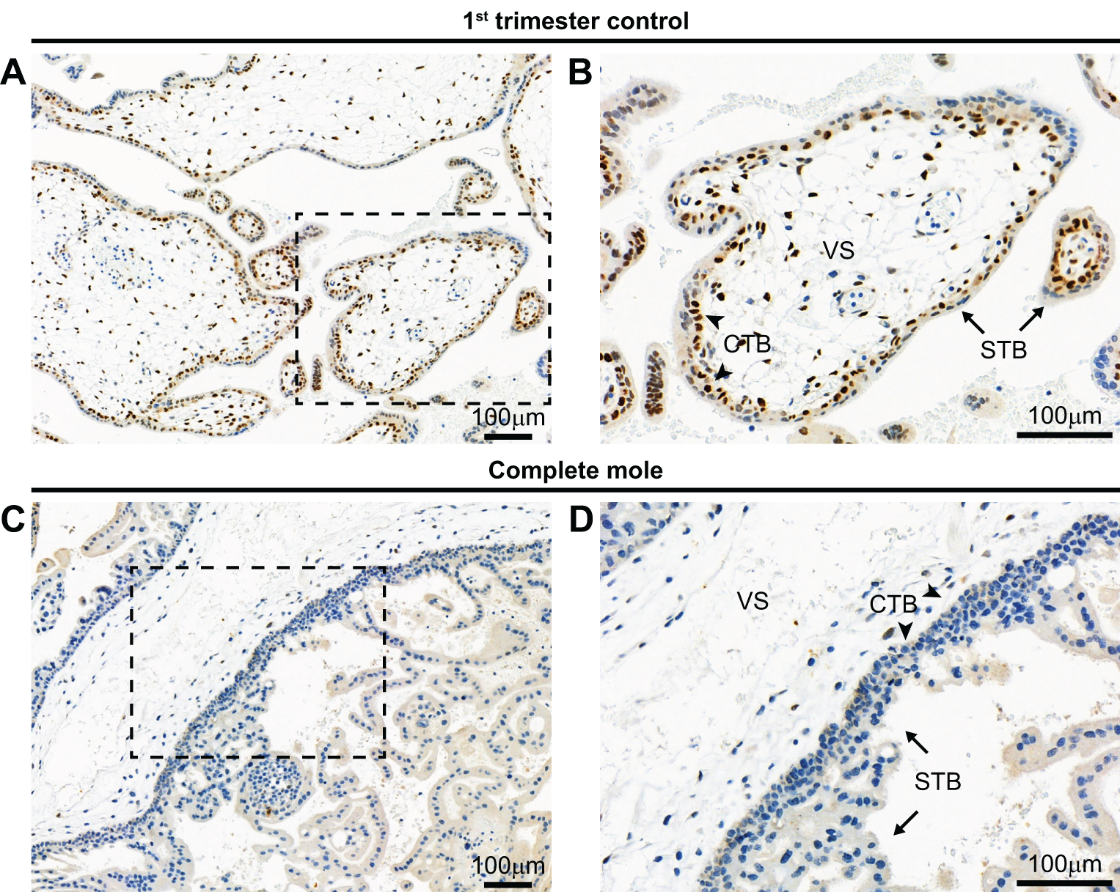


**Supplementary Figure 1.** Differential expression of p57 in cytotrophoblasts in complete hydatidiform moles (CHMs) and first trimester placentas (control). Five-μm-thick first trimester placental sections from normal pregnancy (A-B) or from CHMs (C-D) were stained for p57. Chorionic villi exhibited intense cytotrophoblast nuclear staining (arrowheads), and the syncytiotrophoblast layer was negative (arrows) in normal placentas. The nuclear p57 staining of cytotrophoblasts is absent in CHM. Representative images, hematoxylin counterstain, 100x (A, C) and 200x (B, D) magnifications. Cytotrophoblast, CTB; syncytiotrophoblast, STB; villous stroma, VS.
